# Enhancing the stability of continuous fermentations for platform chemical production

**DOI:** 10.1016/j.isci.2025.111786

**Published:** 2025-01-30

**Authors:** Victoria Outram, Andrew Yiakoumetti, Charlotte Green, Rebekah King, John M. Ward, Alex Conradie

**Affiliations:** 1 Sustainable Process Technologies, Faculty of Engineering, University of Nottingham, University Park, Nottingham NG7 2RD, UK; 2 Department of Biochemical Engineering, University College London, Bernard Katz Building, Gower Street, London WC1E 6BT, UK

**Keywords:** Engineering, Chemistry, Chemical Engineering

## Abstract

A significant bottleneck of continuous fermentation is the stability of plasmid-based expression systems. This work tested five plasmid addiction systems based on essential gene complementation in continuous *E. coli* fermentation. The essential genes tested were *infA*, *ssb*, *proBA*, *proC*, and *dapD* and evaluated under phosphate-limited continuous fermentation at two dilution rates (0.033 h^−1^ and 0.1 h^−1^) and two temperatures (30°C and 37°C). Of these, plasmids stabilized by *infA*, *ssb*, and *dapD* complementation were segregationally stable under all operating conditions. While a lower dilution rate decreased structural stability, this could be remedied by lowering the temperature. At 0.033 h^−1^ and 30°C, addiction systems based on *proC*, *dapD*, and *infA* complementation conferred segregational stability with no detriment to structural stability, enabling higher yields at lower dilution rates. This work expands the potential of continuous fermentations for bio-based platform chemical production using plasmid addiction systems to ensure plasmid stability.

## Highlights

• A comparative characterization of 5 plasmid addiction systems in continuous fermentation
• Decreasing the temperature increases the structural and segregational stability
• Identified robust addiction systems enabling higher yields at lower dilution rate
• Promotes continuous fermentation for the decarbonization of platform chemicals

## Graphical abstract

**Figure:**
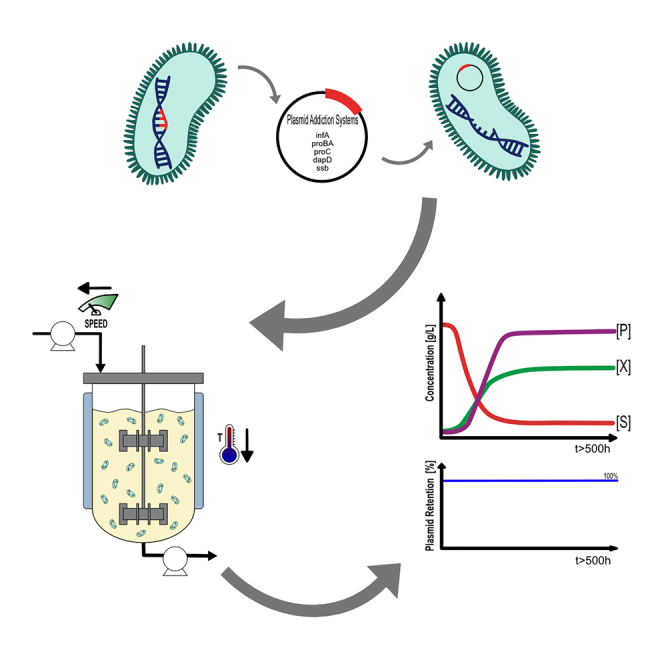

## Teaser

Biotechnology; Chemical Engineering

## Introduction

The deployment of industrial biotechnology has the potential to significantly contribute to improving the global sustainability of the chemical industries by using renewable feedstocks. Microbial fermentation processes were once a staple of the chemical manufacturing industry but were unable to remain economically competitive with current petrochemical manufacturing methods which utilize continuous and intensified processing methods.^1^^,^^2^ Given that microbial fermentations can be exploited to selectively produce a wide range of platform chemicals from renewable feedstocks,^1^^,^^3^ the development of competitive bio-based processes is gaining momentum.

Currently, most industrial bioprocesses utilize batch or fed-batch fermentations.^4^^,^^5^ These operating modes entail substantial turn-around times between batches, representing time windows where the capital invested in the bioreactor is idle and not exploited productively. In contrast, continuous processing will (1) increase the bioreactor volumetric productivity over the total cycle time due to fewer turn-around windows; (2) allow greater product throughput to downstream processing per bioreactor volume; and (3) reduce operational cycling which decreases equipment wear.^6^ These advantages will reduce the capital and maintenance costs.^7^ Additionally, continuous fermentation allows for increased product quality due to steady state operation, enhances integration of the upstream bioprocess with downstream processing, and requires less complex process control.^7^^,^^8^ However, several well-documented challenges are often cited as preventing the widespread deployment of continuous fermentation. These include lower product concentration, increased contamination risk, and the generational instability of microbial production strains under continuous fermentation conditions.^4^^,^^7^^,^^8^

Lower product concentration and the increased contamination risk can be mitigated operationally via the primary recovery unit operation and good aseptic operating procedures. However, the poor stability of microbial production strains is a fundamental barrier to realizing the advantages posed by continuous fermentation. Genetic stability can be defined as segregational or structural stability, where segregational stability is associated with the cell retaining the plasmid, and structural stability is associated with the genetic sequence of the plasmid remaining unchanged by mutations.^9^ For laboratory scale fermentations, plasmid segregational stability is achieved by incorporation of an antibiotic resistance marker into the plasmid and supplementing the corresponding antibiotic to the cell culture. This maintains a selection pressure that prevents the proliferation of plasmid-free cells.^9^ However, the use of antibiotics for commercial scale production is prohibitive due to the cost and environmental concerns,^9^^,^^10^ and so antibiotic-free selection strategies are crucial to maintaining productive continuous fermentations at industrial scales.

One biological strategy is the use of a plasmid addiction system, whereby the plasmid addiction system prevents the survival of non plasmid-bearing cells.^9^ A common method to implement a plasmid addiction system is to delete an essential gene from the chromosome and to express it on the plasmid being stabilized instead. Thereby, cells that have lost the plasmid, via segregational instability, would be unable to proliferate. Examples of addiction systems based on essential gene complementation that have been previously evaluated are summarized in Table 1. Of these addiction systems, only three have been demonstrated in continuous fermentation: *infA*, which demonstrated stability only under phosphate limited conditions;^11^ *trp1*, which proved to be segregationally unstable owing to perceived cross-feeding^12^; and *ssb*, which was only tested for 150 h.^13^ A comprehensive investigation into the performance of plasmid addiction systems based on essential gene complementation is warranted to understand both segregational and structural stability.

**Table 1:** Previous work evaluating essential gene plasmid addiction systems

Essential Gene	Function	Demonstrated in fermentation?	Key fermentation findings	Reference
*infA*	Translation initiation factor 1	Continuous^11^	Only stable under phosphate-limitation.	Yiakoumetti et al.^11^; Hägg et al.^14^
*proBA*	Proline biosynthesis	Batch	No fermentation data provided.	Fiedler et al.^15^
*proC*	Proline biosynthesis	Fed-batch	Part of a two-plasmid system. No plasmid-free cell accumulation, therefore no cross-feeding – not independently tested.	Schneider et al.^16^
*pyrF*	Pyrimidine biosynthesis	Fed-batch	Part of a two-plasmid system. No plasmid-free cell accumulation, therefore no cross-feeding – independently tested. *pyrF was* used as a counter-selection marker.	Schneider et al.^16^
*ssb*	Required for DNA replication and cell viability	Continuous	150 h stability maintained. No segregational stability.	Porter et al.^13^
*trp1*	Tryptophan biosynthesis	Continuous	Cross-feeding observed, therefore not segregationally stable.	DiBiasio et al.^12^
*dapD*	LL-2,6-Diaminopimelate production, lysine biosynthesis.	No	–	Degryse^17^

Independent of plasmid addiction systems, process strategies have also been explored with the aim of improving plasmid stability. Fermentation operating parameters that are typically considered include: selection of the limiting substrate,^18^^,^^19^^,^^20^ fermentation temperature,^21^^,^^22^^,^^23^^,^^24^ and dilution rate.^20^^,^^21^^,^^25^^,^^26^^,^^27^

The biomass concentration in a continuous fermentation is often controlled through the selection of a limiting substrate. In most cases, fermentations are performed under carbon limitation, as the carbon feedstock is a dominant operating cost.^28^ Alternatively, a continuous fermentation could be limited through a nutrient limitation such as phosphate, nitrogen, sulfate, or oxygen limitation. There has been limited comparative work investigating different nutrient limitation strategies; however, it is hypothesized that the limiting nutrient providing the most robust stability may be plasmid dependent.^11^^,^^18^

Previous studies have concluded that increasing the dilution rate increases the plasmid segregational stability. This has been demonstrated across a range of organisms and plasmids, e.g., *E. coli*,^18^^,^^21^^,^^26^ *Saccharomyces cerevisiae*,^12^^,^^25^ *Bacillus stearothermophilus*,^21^ and *Lactococcus lactis*.^27^ The lowest dilution rate that was investigated is 0.1 h^−1^ and 0.7 h^−1^ highest.^12^^,^^25^^,^^27^ Although higher dilution rates provide higher specific productivities, higher rates of biomass turn-over negatively impact the yield. Lower dilution rates increase the yield, as observed by Pejin and Razmovski,^29^ Chen et al.^30^ and Kilonzo et al.*,*^25^ by increasing the carbon flux to product and increase product titers due to slower dilution by fresh media which is preferential for downstream processing.

Similarly, several studies have investigated the impact of temperature on plasmid stability. There is no consensus on the effect of temperature on stability, although most studies concluded that decreasing the temperature increased segregational stability. Sayadi et al.^22^ demonstrated that an *E. coli* continuous culture with a plasmid expressing the *xylE* gene and no antibiotics, exhibited a slower rate of plasmid loss per cell generation (segregational instability) at 31°C compared to 37°C and 42°C. In a similar study, Caulcott et al.^18^ surmised that the growth rate advantage of plasmid-free cells over plasmid-bearing cells is decreased at lower temperatures. Wu et al.^21^ investigated an *E. coli* continuous fermentation at a dilution rate of 0.15 h^−1^ using a plasmid containing the *hok/sok* toxin anti-toxin addiction system. They found that decreasing the temperature from 37°C to 30°C reduced the rate of structural instability from every 15 generations to every 34 generations. Therefore, they also concluded that reducing the temperature slows the cell metabolism, resulting in a lower growth rate advantage over plasmid-free cells. Other works by Son et al.^31^ using *Bacillus megaterium* and Aiba and Koizumi^24^ using *Bacillus stearothemophilus* also found that decreasing the temperature improved segregational stability. In contrast, Craynest et al.^23^ found that 37°C conferred greater segregational stability than 30°C for *Bacillus subtilis.* In summary, these studies encourage the assessment of the impact of temperature on growth rate, productivity, and stability.

This work is an experimental comparison of plasmid addiction systems in continuous fermentation. Five plasmid addiction systems (based on *infA*, *proBA*, *proC*, *ssb*, and *dapD* complementation) were investigated in *E. coli* phosphate-limited continuous fermentations. Of these plasmid addiction systems only *ssb* and *infA* have been tested in continuous fermentation previously, Table 1. These continuous fermentations were evaluated alongside two benchmark strains, one with no stabilization mechanism (i.e., no antibiotics in the fermentation media) and an antibiotic (tetracycline) stabilized strain, stabilized with the continuous addition of tetracycline. The aim was to demonstrate continuous citramalic acid production with these plasmid-based systems maintaining a high steady state specific productivity and yield for 500 h. Citramalic acid is considered non-toxic to *E. coli* tolerating at least 82 g L^−1^,^32^ making a good demonstration chemical, and is of commercial relevance as it can be used as a precursor to other industrial chemicals such as methacrylic acid and methyl methacrylate.^32^^,^^33^ Secondly, this work demonstrates how improvements to both segregational and structural stability were achieved through changing process conditions (dilution rate, D, and temperature, T), thereby maintaining an industrially relevant process. Lastly, this work uses multivariate data analysis to make a comparative assessment across the five addiction systems tested.

## Results

Three different fermentation conditions were tested for seven different strains. Two of these strains, CBC-unstabilized and CBC-tet, are benchmark strains. CBC-unstabilized harbors plasmid pCBC-cam, which expresses a chloramphenicol resistance marker for plasmid maintenance in seed cultures, but fermentations were not supplemented with any antibiotics. Strain CBC-tet harbors plasmid pCBC-tet, which expresses a tetracycline resistance marker, and all fermentations with this strain were performed with tetracycline supplementation to ensure plasmid maintenance. The other five strains (CBC-infA, CBC-proBA, CBC-proC, CBC-ssb, and CBC-dapD), harbored plasmids stabilized via *infA*, *proBA*, *proC*, *ssb*, and *dapD* complementation, respectively. No antibiotics were added to fermentations of any of these strains.

**Figure 1:**
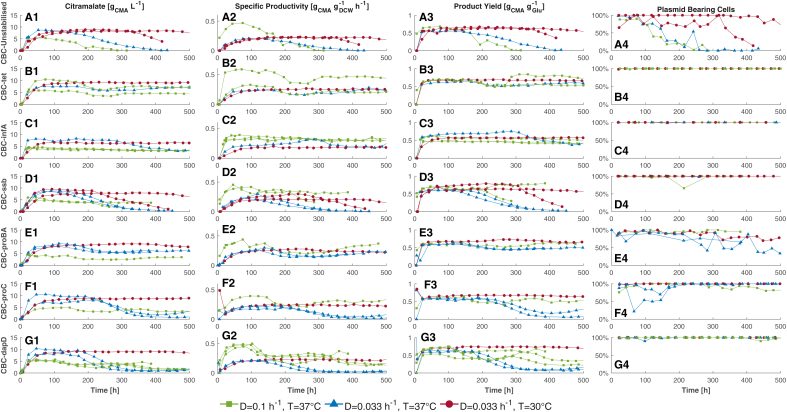
Comparison of continuous fermentations for five test strains and two benchmark strains at the three evaluated operation conditions The columns detail the (1) citramalate concentration, (2) specific productivity, (3) product yield and (4) % plasmid-bearing cells. The rows represent the fermentations for each of the strains in the order of (A) unstabilized strain, (B) tetracycline stabilized, (C) *infA* stabilized, (D) *ssb* stabilized, (E) *proBA* stabilized, (F) *proC* stabilized and (G) *dapD* stabilized, respectively. Red diamonds represent operation at D = 0.033 h^−1^ and T = 30°C. Blue upward triangles represent operation at D = 0.033 h^−1^ and T = 37°C. Green squares represent operation at D = 0.1 h^−1^ and T = 37°C. Each line represents one fermentation at that condition, repeats were performed to confirm the observed instability. CBC-infA at D = 0.1 h^−1^ and T = 37°C is reproduced from prior work.^11^

A dilution rate of 0.1 h^−1^ and an operating temperature of 37°C were used to set a baseline for strain stability based on previous work using CBC-infA.^11^ The impact of reducing the dilution rate from 0.1 h^−1^ to 0.033 h^−1^ at 37°C on yield was investigated, followed by a reduction in operating temperature from 37°C to 30°C at a dilution rate of 0.033 h^−1^ to improve the stability. The continuous fermentation results are summarized in Figure 1. The data presented is based on time rather than number of generations, as the focus of this paper is on optimizing for duration of continuous fermentations rather than investigating the generational stability of the culture. As the biomass concentrations were constant in all fermentations at approximately 1 g L^−1^, and had an excess of glucose, it was assumed that the fermentation nutrient limiting. The media had approximately 30× reduction in phosphate concentration compared to the standard media recipe, with no reduction to other media components, therefore it is assumed phosphate was the only limiting nutrient (supplementary information, Figure S2). Table 2 presents the stability duration (total fermentation time minus the time to reach steady-state) data for the continuous fermentations, along with key fermentation metrics, which are taken as the average across the stable production period of each fermentation.

**Table 2:** Individual fermentation metrics, summarizing the generational stability duration and the fermentation performance metrics during the steady state period of the fermentation

Ferm Ref	Strain	Dilution rate	Temperature	Total duration	Stability duration	[CMA]	Specific Productivity	Specific uptake	Yield (P/S)	Yield (P/X)	Yield (X/S)
	CBC-	h^−1^	°C	h	h	g_CMA_ L^−1^	g_CMA_ g_DCW_^−1^ h^−1^	g_Glu_ g_DCW_^−1^ h^−1^	g_CMA_ g_Glu_^−1^	g_CMA_ g_DCW_^−1^	g_DCW_ g_Glu_^−1^
F1	tet	0.033	37	500	453	6.68	0.21	0.37	0.57	6.42	0.09
F2	tet	0.033	37	500	479	7.97	0.23	0.39	0.58	6.89	0.09
F3	tet	0.033	30	500	449	8.99	0.25	0.37	0.67	7.50	0.09
F4	tet	0.1	37	500	447	4.71	0.41	0.59	0.71	4.12	0.18
F5	proBA	0.033	37	430	388	6.09	0.18	0.36	0.50	5.45	0.09
F6	proBA	0.033	37	500	479	6.88	0.21	0.40	0.52	6.37	0.08
F7	proBA	0.033	30	401	308	8.74	0.25	0.36	0.70	7.67	0.09
F8	proBA	0.1	37	476	456	3.42	0.30	0.51	0.59	3.03	0.20
F9	ssb	0.033	37	166	123	8.14	0.23	0.39	0.60	7.04	0.09
F10	ssb	0.033	37	113	73	8.08	0.20	0.33	0.22	6.04	0.10
F11	ssb	0.033	30	239	171	8.96	0.26	0.35	0.75	7.87	0.10
F12	ssb	0.033	30	406	340	7.60	0.20	0.32	0.63	6.11	0.10
F13	ssb	0.1	37	214	192	4.65	0.35	0.56	0.63	3.54	0.18
F14	ssb	0.1	37	410	384	4.07	0.34	0.49	0.71	3.43	0.21
F15	infA	0.033	37	309	287	7.54	0.24	0.34	0.69	7.16	0.10
F16	infA	0.033	30	499	430	6.38	0.18	0.31	0.57	5.41	0.10
F17	infA	0.1	37	500	475	3.34	0.32	0.72	0.44	3.16	0.14
F18	infA	0.1	37	500	475	3.21	0.31	0.73	0.43	3.13	0.14
F19	infA	0.1	37	500	484	3.74	0.33	0.61	0.55	3.34	0.17
F20	dapD	0.033	37	169	122	9.72	0.20	0.34	0.60	6.17	0.10
F21	dapD	0.033	37	172	148	7.44	0.18	0.28	0.62	5.37	0.12
F22	dapD	0.033	30	451	401	8.84	0.22	0.30	0.73	6.60	0.11
F23	dapD	0.1	37	287	263	4.29	0.33	0.73	0.45	3.33	0.14
F24	dapD	0.1	37	311	284	4.26	0.36	0.55	0.64	3.59	0.18
F25	dapD	0.1	37	127	103	5.20	0.41	0.60	0.68	4.13	0.17
F26	proC	0.033	37	191	144	9.96	0.21	0.37	0.58	6.38	0.09
F27	proC	0.033	37	239	196	7.27	0.17	0.30	0.56	5.16	0.11
F28	proC	0.033	30	500	449	8.59	0.23	0.35	0.66	7.05	0.09
F29	proC	0.1	37	500	460	3.85	0.30	0.56	0.53	3.02	0.18
F30	unstabilized	0.033	37	145	125	7.63	0.17	0.31	0.54	5.09	0.12
F31	unstabilized	0.033	30	337	242	8.51	0.22	0.34	0.65	6.71	0.10
F32	unstabilized	0.033	30	451	385	7.98	0.21	0.35	0.60	6.41	0.09
F33	unstabilized	0.1	37	74	50	5.32	0.43	0.65	0.66	4.29	0.15

### Stability baseline

**Figure 2:**
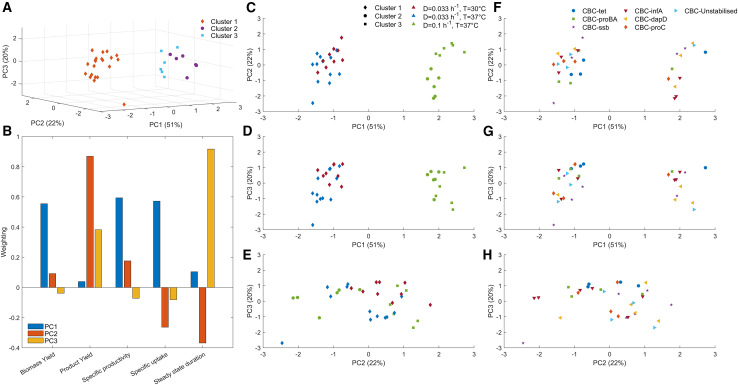
PCA of the steady state fermentation data for the first three principal components explaining 93% of the variance (A) Shows the 3D data with k-means clustering. (B) Component weightings for each PC. (C–E) 2D projections of PCs, with color representing fermentation conditions and shape distinguishing the k-means cluster. (F–H) 2D projections of PCs, with shape and color denoting the seven different strains.

**Figure 3:**
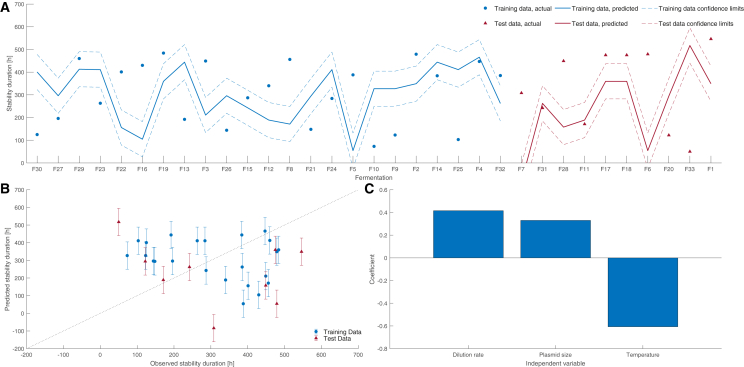
Multiple linear regression model for the fermentation data (A) Evaluation of training and testing data, 95% confidence limits; (B) demonstrates the model fit, the bars indicate the 95% confidence limit of the model; (C) the weighted contribution of the independent variables.

**Figure 4:**
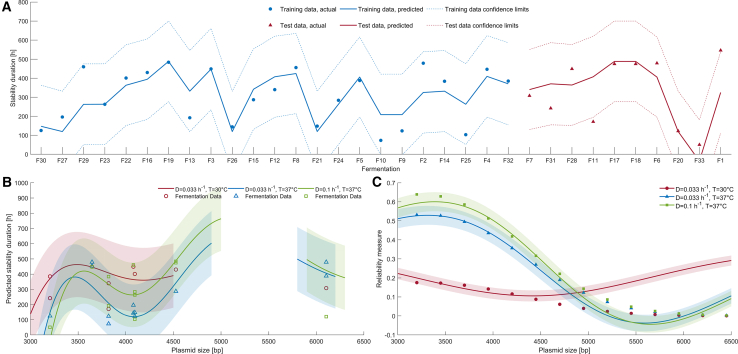
Radial basis function model for the fermentation data (A) Regression results for the training data (blue) and the prediction using the test data (red), with dashed lines representing 95% confidence limits. (B) Predicting the generational stability based on plasmid size, for the three fermentation operating conditions. The 95% confidence limits for each prediction are represented by the corresponding shaded region, interrupted to also reflect the reliability limits. The open shapes show the actual fermentation data. (C) Demonstrating the reliability of the predicted data, where points outside the 95% confidence limits are designated as uncertain extrapolation.

First, the segregational and structural stability of the two benchmark strains, CBC-unstabilized and CBC-tet, were assessed in continuous fermentation at a dilution rate of 0.1 h^−1^ and an operating temperature of 37°C. This set a performance baseline in the absence of addiction systems. From Figure 1, Figure 2, Figure 3, Figure 4-A1, the percentage of plasmid-bearing cells for CBC-unstabilized decreased markedly 24 h after inoculation, mirrored by a correlated decrease in titer (Figure 1-A1), specific productivity (Figure 1, Figure 2-A2) and yield (Figure 1, Figure 2, Figure 3-A3). Poor segregational stability dominated the outcome of these fermentation output parameters. Conversely, CBC-tet maintained 100% plasmid bearing cells in a continuous fermentation for over 500 h (Figure 1-B4) with patch plating demonstrating all colonies tested had retained the plasmid. However, long period oscillations were evident in both biological repeats in the product titers and even more evident in the productivity and yield data due to similar fluctuations in the residual glucose concentration, Figure 1-B1 and B2. Similar oscillatory behavior was evident for the fermentations of strains CBC-proBA, CBC-proC, and CBC-dapD. There was 100% plasmid retention in the fermentation of strain CBC-dapD, though there was a stepwise decline in productivity which most likely indicates structural instability in the three biological repeats. Notably, at a dilution rate of 0.1 h^−1^, both CBC-proBA and CBC-proC were likely susceptible to cross-feeding due to a proportion of plasmid-free cells being present in the fermentation, as observed in Figure 1-E4 and Figure 1-F4, respectively. Interestingly, there is no corresponding decrease in citramalate concentration, as shown in Figure 1-E1 and 1-F1. For CBC-ssb and CBC-infA, there was no observable oscillatory behavior. Both these addiction systems demonstrated segregational stability. However, structural instability was observed in one of the biological repeats of CBC-ssb, evident through the decrease in product concentration. The fermentation of strain CBC-infA maintained a steady state citramalate concentration, indicating segregational and structural stability throughout, as discussed in prior work.^11^

### Stability at a lower dilution rate

The dilution rate was decreased to 0.033 h^−1^ in an attempt to increase the product yield based on the results of Pejin and Razmovski,^29^ Chen et al.,^30^ and Kilonzo et al*.*^25^ In the absence of selection pressure, the decline in plasmid retention during the fermentations of CBC-unstabilized with respect to fermentation time was comparable to that in fermentations performed at a dilution rate of 0.1 h^−1^. Due to the reduction in dilution rate by a factor of 3, the resulting instability occurred in fewer generations, which agrees with other studies in the field using *E. coli* as production strain.^18^^,^^21^^,^^22^^,^^26^ For CBC-tet, the fermentation performance was comparable to that performed at a dilution rate of 0.1 h^−1^ including similar oscillatory behavior, with no appreciable increase in yield or decrease in specific productivity. Segregational instability due to potential cross-feeding for CBC-proBA was more pronounced at the lower dilution rate, resulting in approximately only 40% of the cell population containing the plasmid. However, the fermentation performance was only decreased marginally despite a substantial loss of plasmid-bearing cells as evidenced by the two biological repeats. The dynamic behavior for CBC-proBA fermentations remained oscillatory, exhibiting a long, shallow periodicity. Sustained cross-feeding was not evident in CBC-proC (Figure 1-F4) since both biological repeats demonstrated a recovery in plasmid-bearing cells to 100% segregational stability. However, the dynamic behavior shifted from oscillatory to structural instability. At lower dilution rate, CBC-dapD maintained 100% segregational stability; however, both biological repeats showed pronounced structural instability compared to 0.1 h^−1^. Similarly, both CBC-ssb biological repeats were impacted by structural instability, as no segregational stability was observed. CBC-infA was the only strain to demonstrate the desired increase in product yield at a lower dilution rate. However, after 290 h, structural instability was observed resulting in a decrease in productivity.

Overall, a decrease in dilution rate from 0.1 h^−1^ to 0.033 h^−1^ increased the likelihood of instability. Only CBC-tet, which was the only strain subjected to an external selection pressure, maintained a similar performance at both dilution rates. This is potentially due to tetracycline having antioxidant potential, and therefore reducing the concentration of reactive oxygen species (ROS) in the cytosol. As ROS are mutagenic, reducing the concentration of ROS might reduce the likelihood of a deleterious genetic mutation during the time frame of the fermentation.^11^^,^^34^

### Stability at a lower temperature

Process solutions were implemented to increase the stability at low dilution rate, by decreasing the operational temperature to 30°C, as demonstrated by Wu et al*.*^21^ This eliminated the oscillatory dynamic behavior observed at 37°C. At 30°C, structural stability was finally established with CBC-dapD. Segregational instability was also reduced for CBC-proBA compared to when operating fermentations at T = 37°C and D = 0.033 h^−1^. Moreover, segregational instability was eliminated for CBC-proC, and structural stability was improved too. For all strains, the highest product yield was observed (Figure 1-E3, F3, and G3). Structural stability was re-established for CBC-infA at lower productivity than at 37°C and 0.1 h^−1^. Remarkably, CBC-unstabilized demonstrated >90% segregational stability for one of the two biological repeats over 500 h, and >75% in the other repeat for 320 h before citramalate production collapsed, as shown in Figure 1-A4 and A1. Only CBC-ssb exhibited structural instability at 30°C, and this was for one of the two biological repeats. CBC-tet maintained segregational and structural stability over a period of 500 h and exhibited improved product yield at 30°C.

Other than for CBC-ssb, at 30°C the likelihood of segregational or structural instability stability was reduced. Overall, reducing the temperature has a positive effect on the fermentations. At D = 0.033 h^−1^, transitioning from 37°C to 30°C resulted in a 20% increase in the average yield from 0.55 to 0.66 g_CMA_ g_Glu_^−1^, and a 9% increase in the average specific productivity from 0.20 to 0.22 g_CMA_ g_DCW_^−1^ h^−1^.

### Multivariate data analysis

The incomplete mechanistic understanding of most biological systems increases the complexity in interpreting fermentation data with respect to the strain design. It is possible to utilize advanced multivariate data analysis (MVDA) from historical data to identify patterns and make informed inferences about the fermentation. In fermentation processes MDVA is typically used for characterizing batch to batch variability and for time-variant process monitoring and fault detection.

Within this work, there is a high degree of inherent variance due to the plasmid design, (i.e., addiction system, plasmid backbone, promoters and ribosome binding sites, as shown in Table 3) and fermentation operation, a high degree of variability was anticipated. To better understand this variability and account for it, the data presented in Table 2 were subject to both a principal component analysis and regression analysis.

**Table 3:** Summary of the essential gene stabilized plasmid design highlighting the variability between strains (Cam = chloramphenicol, Tet = tetracycline)

Strain	Plasmid	Origin of replication (derived from plasmid)	Antibiotic resistance marker	Addiction marker	Addiction gene promoter^b^	RBS for plasmid stabilization	CMA promoter
CBC-infA^11^	pCBC-9	pMB1 (but does not include rop, as amplified from pBAD24)	Cam	*infA*	N/A *infA* was expressed in an operon with Cam resistance marker	Native/Amplified from chromosomes^c^	J23104
CBC-proBA	pCBC-V33	pCOLA (amplified from pCOLADuet™-1)^a^	Tet	*proBA*	Tet promoter	Native/Amplified from chromosomes^c^	J23119
CBC-ssb	pCBC-V36	pCOLA (amplified from pCOLADuet™-1)^a^	Cam	*ssb*	Native	Native/Amplified from chromosomes^c^	J23119
CBC-proC	pCBC-V43:proC	pCOLA (amplified from pCOLADuet™-1)^a^	Cam	*proC*	mob	Native/Amplified from chromosomes^c^	J23119
CBC-dapD	pCBC-V43:dapD	pCOLA (amplified from pCOLADuet™-1)^a^	Cam	*dapD*	mob	Native/Amplified from chromosomes^c^	J23119
CBC-tet	pCBC-V24P1	pCOLA (amplified from pCOLADuet™-1)^a^	Tet	N/A	N/A	N/A	J23119

a The pCOLA derived backbone was used in a different study (unpublished data) for a more complex pathway expressed over two plasmids, where two different origins of replication were required. Therefore, this plasmid backbone was also considered in this study.

b The addiction gene promoter was adjusted to ensure the cell was producing sufficient quantities of the addiction gene, to not compromise cell functionality and maintain the plasmid during shake flask cultures.

c See supplementary sequences.

**Table 4:** Strain specific media and cultivation conditions for fermentation

Strain	Gene size (bp)	Plasmid size (bp)	Starter culture/inoculum antibiotics	Fermentation supplementation	Non-selective plates	Selective plates
CBC-unstabilized	0	3205	34 μg mL^−1^ chloramphenicol		MS + glucose	MS + glucose +34 μg mL^−1^ chloramphenicol
CBC-tet	0	3646	12 mg L^−1^ tetracycline	12 mg L^−1^ tetracycline	MS + glucose	MS + glucose +12 mg L^−1^ tetracycline
CBC-infA	219	4526	34 μg mL^−1^ chloramphenicol		MS + glucose	MS + glucose +34 μg mL^−1^ chloramphenicol
CBC-ssb	537	3820	34 μg mL^−1^ chloramphenicol		MS + glucose	MS + glucose +34 μg mL^−1^ chloramphenicol
CBC-proBA	2358	6104			MS + glucose +100 μg L^−1^ L-proline	MS + glucose
CBC-proC	810	4080			MS + glucose +100 μg L^−1^ L-proline	MS + glucose
CBC-dapD	825	4095			MS + glucose +250 μg L^−1^ DAP	MS + glucose

See data availability statement for plasmid maps.

#### Principal component analysis

For fermentation data, principal component analysis (PCA) is the dominant multivariate data analysis method used and has been demonstrated for comparative analysis of both batch and fed-batch fermentation data at laboratory scale^35^^,^^36^ and industrial scale.^37^^,^^38^^,^^39^^,^^40^^,^^41^ These applications are predominantly geared toward process monitoring (supervision) and fault detection, classification, and the creation of on-line inferential (soft) sensors. Only Yang et al.^36^ utilized PCA in support of process development, assessing eight physicochemical properties from nine small scale continuous anaerobic digesters.

A PCA was undertaken on the five fermentation performance parameters: stability duration, biomass yield, citramalate yield, specific glucose uptake rate, and specific citramalate productivity. The aim of the PCA was to ascertain whether the performance parameters would implicitly cluster along the experimental design factors: addiction system type, dilution rate and temperature. The PCA reduced the performance parameter dimensionality to three principal components explaining greater than 93% of the total variance. Principal component 1 (PC1) explained 51% of the variance dominated by linear combinations of the specific productivity, specific uptake rate and biomass yield, with approximately equal weighting (Figure 2B). PC2 explained 22% of the variance dominated by a positive correlation with product yield, and a negative correlation with stability duration. PC3 explained 20% of the variance dominated by steady state stability (Figure 2B).

Given the three operating conditions and three experimental design factors, a k-means clustering analysis was performed with the aim of discretising for three clusters. Figures 2C–2E show the projections of the PCA, highlighting the three clusters and the three operating conditions. There is a clear distinction between cluster 1 with clusters 2 and 3, and this is associated with the two different dilution rates tested. This further emphasizes the impact of dilution rate on fermentation performance. With high biological growth rates there is increased carbon flux toward biomass formation, which is competing with the synthesis of the product. Therefore, the concentration of the product is reduced. However, clusters 1 and 3 are only partially separated by temperature. Cluster 3, i.e., PC1 <0 and PC3 <0, could be considered a marker for instability as most of the fermentations included are at T = 37°C and D = 0.033 h^−1^, which provides the least stable operating conditions, plus the two least stable fermentations at T = 30°C and D = 0.033 h^−1^, Table 2 F11 and F31. As the stability duration is the key contributing variable to PC3, the fermentations falling below zero in the third principal component are likely to be unstable. This can be further verified when assessed alongside the addiction system classification of the same data, Figure 2G; whereby at D = 0.1 h^−1^ the fermentations with PC3 <0 are with CBC-unstabilized, CBC-ssb, and CBC-dapD, all of which demonstrated instability at this dilution rate.

Figures 2F–2H show the PCA data color coded according to the plasmid addiction system. Prior to execution of the experimental design, it was postulated that the PCA would have clustered the addiction systems along defined type boundaries, such as cellular process or amino acid biosynthesis, but the addiction systems did not cluster, remaining diffuse across their corresponding dilutions rates. Even PC2 vs. PC3, Figure 2H, which explains 20–22% of the variance each, does not capture nuances between the different addiction systems. This leads to the conclusion that the choice of essential gene does not have a significant impact on the stability of the fermentation, and with optimization of the strain and plasmid, the potential for long-term stable production should not be ruled out.

#### Regression analysis

As the first three principal components explained 93% of the variance and broadly clustered by operating condition, regression analysis was used to understand the impact of the experimental factors (independent variables) to predict the stability duration. As plasmid size has long been associated with plasmid stability,^10^ this was selected as the factor to quantitatively represent the plasmid construct, and therefore addiction system differences between strains.

##### Multiple linear regression

A multiple linear regression (MLR) model was trained using 70% of the standardized fermentation data using dilution rate, plasmid size, and temperature as independent variables. The proportional weighting of each independent variable’s contribution to predicting the stability duration is presented in Figure 3C, supporting the positive and negative correlations with dilution rate and temperature respectively, alongside a weak positive correlation to plasmid size. However, Figure 3A shows the model is not capable of predicting the stability duration, where the majority of the training data are outliers to the model’s predictive interval. Validating the model, only 20% of the test data fell within the 95% confidence limit, where the upper and lower bounds span approximately 155 h. Overall, Figure 3B shows no correlation between the model prediction and the observed data. This indicates that the three independent variables cannot explain the stability duration variance, despite the distinct PCA clustering associated with dilution rate and temperature. Alternatively, the data are non-linear and, consequently, cannot be described using a linear model structure.

##### Radial basis function modeling

Radial basis function modeling (RBF) is one of the simplest forms of an artificial neural network with a single hidden layer.^42^ The hidden layer is formed of radial functions, which are functions with their main characteristic being a monotonic decrease or increase from a central point.^43^ Compared to conventional sigmoidal neural networks, RBFs can provide localized mapping of the input vector space to the output vector space, therefore they can be relied upon to extract features directly from the input space. Orr^43^ and Ghosh and Nag^44^ provide a detailed introduction to RBFs.

Within fermentation, RBF has typically been used for soft sensor development, using online data to predict fermentation performance parameters, such as biomass concentration, yield, and product titer.^44^^,^^45^^,^^46^^,^^47^^,^^48^ Furthermore, radial basis functions are also amenable to the calculation of confidence limits and extrapolation detection, where Warnes et al.^45^ demonstrated the calculation of confidence limits for biomass and product concentration inferential sensors using the methodology proposed by Leonard et al*.*^49^

Given the flexibility of radial basis functions to extract features and produce confidence limits,^45^^,^^46^ this study selected radial basis function neural networks as regression technique to make statistical inferences from the strain development fermentation data. To avoid over-extrapolation, this study implemented a modified reliability measure drawing from Leonard et al.^49^ and Lopes and Menezes.^50^ This was developed using the same three independent variables, training and test data, as for the MLR. Given the first three principal components of the fermentation performance parameters explained 93% of the variance, three hidden nodes were selected to affect implicit feature extraction within the RBF neural network. Figure 4A shows the regression to both the training and test data, including the lower and upper confidence limits of the model. Unlike the MLR, all the observed training data fell within the confidence limits, suggesting no outliers beyond the unexplained variance of the data.

The generalization against the test data was reasonable with 90% of the test data falling within the model’s confidence limits. The enhanced predictive robustness confirms the non-linear relationship between the dependent and independent variables. Nevertheless, the unexplained variance and the limited number of fermentations produced confidence bounds spanning approximately ±200 h, or 8 days of continuous fermentation.

Given the explained variance of the non-linear regression, the RBF model was used to draw generalizable inferences setting aside the unexplained variance. The reliability measure effectively prevented any generalization beyond ±0.5°C and ±0.01 h^−1^ of the fermentation input parameter, limiting the model’s utility to only the tested operating parameters. However, due to the wider range of plasmid sizes within the dataset, the model could be used to infer the impact of plasmid size between 3,000 bp to 4,500 bp and 6,100 ± 100 bp, Figure 4C, where between 4,500 bp and 6,000 bp the reliability measure falls outside of the confidence limits. This could be further bound by a maximum stability duration of 500 h, in line with the bounds of the experimental method used.

The generalized inference from the RBF model supports the observed relationships from the PCA. Decreasing the dilution rate from 0.1 h^−1^ to 0.033 h^−1^ at 37°C decreases the nominal prediction for stability duration. Decreasing the temperature from 37°C to 30 °C at 0.033 h^−1^, increases the nominal prediction for stability beyond that of 0.1 h^−1^ at 37°C. However, the overlapping confidence limits are telling of the unexplained variance and the limited number of fermentations, meaning the RBF model cannot discern between the three fermentation operating conditions. Plausibly, the unexplained variance could stem from plasmid construct differences, such as plasmid backbone, promoters and ribosome binding sites, and fermentation operational variability. This implies that additional independent variables are required to explain a greater proportion of the variance.

## Discussion

Previous work has focused on considerably higher dilution rates that are impractical for large scale fermentations, where 0.1 h^−1^ is typically at the lower end of dilution rates investigated.^12^^,^^25^^,^^27^ For platform chemicals, high product yields are advantageous to supporting a feasible techno-economic outcome.

This study considered two benchmark strains, CBC-unstabilized with no stabilization pressure applied and CBC-tet with antibiotic selection pressure, under three sets of fermentation conditions. Both these strains performed in agreement with previous literature observations, where plasmid segregational instability was observed in fermentations of strain CBC-unstabilized and was exacerbated as the dilution rate decreased. However, decreasing the temperature counteracted this by increasing segregational stability, enabling steady state citramalate production for 385 h.^21^^,^^22^ Tetracycline selection pressure enforced both segregational and structural generational stability across the three operating conditions, noting that antibiotic selection pressure is both environmentally and techno-economically undesirable. The oscillatory behavior that was observed at 37°C has been previously observed in continuous culture^51^^,^^52^ and is typically attributed to nutrient limitation.^19^

Previously, the *dapD* addiction system has been associated with plasmid stability both via a repressor titration system,^53^ and as an essential gene deletion and complementation addiction system,^17^ though the latter has not been demonstrated in fermentation, Table 1. Across the three operating conditions, plasmid-free cells were not maintained in the culture indicating that cross-feeding is unlikely, and therefore segregational stability was maintained. At T = 37°C for both dilution rates, structural instability was evident. However, decreasing the temperature to 30 °C at 0.033 h^−1^, established structural stability for CBC-dapD for 500 h.

For both L-proline based addiction systems, *proBA* and *proC*, potential cross-feeding was evident to varying degrees. Cross-feeding has been previously reported when using an essential gene involved in amino acid synthesis.^9^^,^^13^^,^^14^^,^^16^ At 30°C, the potential cross-feeding for both CBC-proBA and CBC-proC was reduced. As previously stated, reducing the temperature reduces the metabolic activity, therefore this could potentially lead to a reduction in proline production and hence less freely available proline in the fermentation broth for cross-feeding.

Previously, Porter et al.^13^ had demonstrated that the *ssb* addiction system was generationally stable over 150 h of continuous fermentation, which is in agreement with this study given the onset of structural stability typically occurs >150 h. In contrast to the other addiction systems, CBC-ssb demonstrated structural instability under all operating conditions. Though more structurally stable at 30°C, CBC-ssb still suffered from structural instability more readily than the other four addiction systems as evidenced by biological repeats. Given that ssb is involved in DNA repair^54^ it is possible that the deletion of this gene from the chromosome and its expression instead in this study from a sub-optimal constitutive promoter led to its deregulation, resulting in increased mutagenesis rates.

CBC-infA showed consistent fermentation performance, i.e., 100% segregational stability, no oscillatory dynamic behavior at 0.1 h^−1^ and 37°C,^11^ and moderate structural instability at 0.033 h^−1^ and 37°C resulting in gradually decreasing CMA titers. At 0.033 h^−1^ and 30°C, CMA production remained stable, suggesting structural stability of the plasmid.

The PCA confirmed the importance of the process operating conditions on the generational stability in continuous fermentation. The distinct clustering associated with dilution rate and temperature supported a stronger contribution to stability than the selection of a specific addition system where no distinct clustering is apparent. The MLR highlighted the non-linear relationship between stability duration, process conditions and plasmid design. While the non-linear RBF supported the observed relationships from the PCA, the overlapping confidence limits indicate that the model’s independent variables do not explain all the variance in the fermentation data.

Even though the dataset presented here represents a large dataset for fermentation experimentation, additional fermentations are required to enable a more robust statistical analysis. Secondly, it should be highlighted that the fermentations were limited to 500 h. While this exceeds the duration in previously published works with respect to all plasmid addiction systems, other than *infA* (Table 1), these fermentations could have been run for longer. Furthermore, extending the fermentation duration beyond 500 h may allow for greater separation between the plasmid addiction systems performance capability. Additionally, as the experiments were designed to investigate the contribution of plasmid addiction systems to continuous fermentation performance, further experimentation is required to pinpoint the biological mechanisms responsible for the phenotypical observations.

### Conclusion

This work comprehensively tested and compared five plasmid addiction systems (*infA*, *ssb*, *proBA*, *proC*, and *dapD*) in continuous *E. coli* fermentations at two dilution rates (0.033 h^−1^ and 0.1 h^−1^) and two temperatures (30°C and 37°C). Of these, *infA*, *ssb*, and *dapD* were segregationally stable; while *proBA* and *proC*, under some conditions, were not segregationally stable and demonstrated potential cross-feeding. A higher dilution rate increased structural stability, and lowering the temperature also increased the structural stability. At 0.033 h^−1^ and 30°C *proC*, *dapD*, and *infA* are robust addiction systems, both segregationally and structurally, enabling higher yields at lower dilution rates. This work increases the understanding of continuous fermentation for bio-based platform chemical production, demonstrating a range of plasmid addiction systems and process conditions to maintain a stable fermentation for 500 h. This will enable the decarbonization and transition to a circular economy for the manufacturing industry.

### Limitations of the study

This work was limited to two temperatures, two dilution rates and five plasmid addictions, only undertaken in *E. coli*. The five addiction systems selected did not consider toxin/anti-toxin systems or operator-repressor titration systems, instead focusing on essential gene deletion and complementation. Additionally, the fermentations were performed at a low cell density (∼1 g L^−1^). The trends observed between stability, temperature and dilution rate align well with the conclusions for *E. coli* in literature. This study has produced a considerably large fermentation dataset, and is provides evidence of five plasmid addiction systems in continuous fermentation in *E. coli*. This information can be used for a quantitative comparison based on the fermentation metrics. However, as discussed in the discussion (Plasmid addiction system selection), the dataset generated is still not large enough to elucidate the minor differences between the different plasmid addiction systems. Data analysis could be further enhanced with sequencing of the plasmids throughout the fermentations to better understand and pinpoint where instability occurs. Lastly, further work should consider investigation into key plasmid features such as plasmid backbone on the stability of the fermentation.

## Resource availability

### Lead contact

Further information and requests for resources and reagents should be directed to and will be fulfilled by the lead contact, Alex Conradie (a.conradie@ucl.ac.uk).

### Materials availability

• This study did not generate unique reagents.
• Strains and plasmids constructed in this paper can be made available upon request. Requests should be made to the lead contact, Alex Conradie.

### Data and code availability

• Data supporting, including plasmid maps and continuous fermentation data, for this work can be accessed at https://doi.org/10.6084/m9.figshare.23976987.
• This study did not generate original code.
• Any additional information required to reanalyze the data reported in this paper is available from the lead contact upon request.

## Acknowledgments

The authors would like to thank Dr Lifang Zhang for the assistance with patch plating required in this work. This work was supported by Industrial Biotechnology Catalyst project, ConBioChem: Continuous bio-production of commodity chemicals, funded by Innovate UK, 10.13039/501100000268BBSRC and 10.13039/501100000266EPSRC (grant BB/N023773/1).

## Author contributions

V.O., conceptualization, methodology, software, formal analysis, investigation, data curation, writing - original draft, writing - review and editing, and visualization; A.Y., conceptualization, methodology, investigation, writing - review and editing; C.G., conceptualization, methodology, and investigation; R.K., methodology (radial basis function modeling); J.M.W., conceptualization, resources, and writing – review and editing; A.C., conceptualization, methodology, software, formal analysis, resources, writing - review and editing, and supervision.

## Declaration of interests

The authors declare no competing interests.

## STAR★Methods

### Key resources table

**Table:** 

REAGENT or RESOURCE	SOURCE	IDENTIFIER
**Bacterial and virus strains**		

*E. coli* BW25113 ΔldhA::FRT	Yiakoumetti et al.^11^	https://doi.org/10.1016/j.isci.2025.111787

**Chemicals, peptides, and recombinant proteins**		

LB Broth	Sigma	Cat# L3522
Agar	Sigma	CAS 9002-18-0
(NH_4_)_2_SO_4_	Fisher	CAS 7783-20-2
K_2_HPO_4_	Fisher	CAS 7758-11-4
NaH_2_PO_4_·H_2_O	Fisher	CAS 10049-21-5
(NH_4_)_2_H-citrate	Fisher	CAS 3012-65-5
D-Glucose	Fisher	CAS 50-99-7
HCl	Fisher	CAS 7647-01-0
Na_2_EDTA·2H_2_O	Fisher	CAS 6381-92-6
CaCl_2_·2H_2_O	Fisher	CAS 10035-04-8
FeCl_3_	Fisher	CAS 7705-08-0
ZnSO_4_·7H_2_O	Fisher	CAS 7446-20-0
CuSO_4_·5H_2_O	Fisher	CAS 7758-99-8
MnSO_4_·H_2_O	Fisher	CAS 10034-96-5
CoCl_2_·6H_2_O	Fisher	CAS 7791-13-1
KH_2_PO_4_	Fisher	CAS 7778-77-0
NH_4_Cl	Fisher	CAS 12125-02-9
MgSO_4_·7H_2_O	Fisher	CAS 10034-99-8
KOH	Fisher	CAS 1310-58-3
CaCl_2_	Fisher	CAS 10043-52-4
MnCl_2_·4H_2_O	Fisher	CAS 13446-34-9
FeSO_4_.7H_2_O	Fisher	CAS 7782-63-0
(NH_4_)_6_Mo_7_O_24_·4H_2_O	Fisher	CAS 12054-85-2
Polypropylene glycol 2000	Fisher	CAS 25322-69-4
L-proline	Sigma	CAS 147-85-3
LL-2,6-diaminopimelic acid	Sigma	CAS 583-93-7
Chloramphenicol	Sigma	CAS 56-75-7
Tetracycline	Sigma	CAS 60-54-8

**Critical commercial assays**		

Q5 DNA polymerase	New England Biolabs UK Ltd	M0491
NEBuilder® HiFi DNA Assembly Master Mix	New England Biolabs UK Ltd	E2621
*Bam*HI (*Bam*HI-HF variant)	New England Biolabs UK Ltd	R3136S
*Sbf*I (*Sbf*I-HF variant)	New England Biolabs UK Ltd	R3642S
*Bgl*II	New England Biolabs UK Ltd	R0144S
*Sap*I	New England Biolabs UK Ltd	R0569S
T4 DNA Ligase	New England Biolabs UK Ltd	M0202S
GenElute™ Bacterial Genomic DNA Kit	Sigma Aldrich	NA2110

**Deposited data**		

Plasmid & strain construction: plasmid maps	This study	Figshare: https://doi.org/10.6084/m9.figshare.23976987
Continuous fermentation data	This study	Figshare: https://doi.org/10.6084/m9.figshare.23976987

**Experimental models: Organisms/strains**		

*E. coli* BW25113 Δ*ldhA*::FRT Δ*proBA*::sp^R^	This study	N/A
*E. coli* BW25113 Δ*ldhA*::FRT Δ*proC*::sp^R^	This study	N/A
*E. coli* BW25113 Δ*ldhA*::FRT Δ*dapD*::sp^R^	This study	N/A
*E. coli* BW25113 Δ*ldhA*::FRT Δ*ssb*::*sp*^*R*^	This study	N/A
CBC-tet: *E. coli* BW25113 Δ*ldhA*::FRT pCBC-V24P1	This study	N/A
CBC-proBA: *E. coli* BW25113 Δ*ldhA*::FRT Δ*proBA*::*sp*^*R*^ pCBC-V33	This study	N/A
CBC-ssb: *E. coli* BW25113 Δ*ldhA*::FRT Δ*ssb*::*sp*^*R*^ pCBC-V36	This study	N/A
CBC-proC: *E. coli* BW25113 Δ*ldhA*::FRT Δ*proC*::*sp*^*R*^ pCBC-V43:*proC*	This study	N/A
CBC-dadD: *E. coli* BW25113 Δ*ldhA*::FRT ΔdapD:*sp*^*R*^ pCBC-V43:*dapD*	This study	N/A
CBC-infA: *E. coli* BW25113 Δ*ldhA*::FRT Δ*infA*::km^R^ pCBC-9	Yiakoumetti et al.^11^	CBC-9

**Oligonucleotides**		

Oligonucleotides used are in Table S4	This study	N/A

**Recombinant DNA**		

Gblock-1 gene fragment: ACTATTTGCAACAGTGCCGTTGATCGTGCTATGATCGA CTGATGTCATCAGCGGTGGAGTGCAATGTCATGAGGG AAGCGGTGATCGCCGAAGTATCGACTCAACTATCAGA GGTAGTTGGCGTCATCGAGCGCCATCTCGAACCGACG TTGCTGGCCGTACATTTGTACGGCTCCGCAGTGGATG GCGGCCTGAAGCCACACAGTGATATTGATTTGCTGGT TACGGTGACCGTAAGGCTTGATGAAACAACGCGGCG AGCTTTGATCAACGACCTTTTGGAAACTTCGGCTTCCC CTGGAGAGAGCGAGATTCTCCGCGCTGTAGAAGTCAC CATTGTTGTGCACGACGACATCATTCCGTGGCGTTAT CCAGCTAAGCGCGAACTGCAATTTGGAGAATGGCAG CGCAATGACATTCTTGCAGGTATCTTCGAGCCAGCCA CGATCGACATTGATCTGGCTATCTTGCTGACAAAAGCA AGAGAACATAGCGTTGCCTTGGTAGGTCCAGCGGCG GAGGAACTCTTTGATCCGGTTCCTGAACAGGATCTATT TGAGGCGCTAAATGAAACCTTAACGCTATGGAACTCG CCGCCCGACTGGGCTGGCGATGAGCGAAATGTAGTG CTTACGTTGTCCCGCATTTGGTACAGCGCAGTAACCG GCAAAATCGCGCCGAAGGATGTCGCTGCCGACTGGG CAATGGAGCGCCTGCCGGCCCAGTATCAGCCCGTCA TACTTGAAGCTAGACAGGCTTATCTTGGACAAGAAGAA GATCGCTTGGCCTCGCGCGCAGATCAGTTGGAAGAAT TTGTCCACTACGTGAAAGGCGAGATCACCAAGGTAGT CGGCAAATAA	Sophie Vaud	Gblock-1
PCR Fragment: Amplified from plasmid pCOLADuet-1 using primers V24.oriColA.F & V24.oriColA.R	pCOLADuet-1	V24.1
PCR Fragment: Amplified from plasmidpCOLADuet-1 using primers V24.ColATermT7.F & V24.ColATermT7.R	pCOLADuet-1	V24.2
PCR Fragment V24.3: Amplified from plasmidpSC101 using primers V24.TcR.F & V24.TcR.R	This study	N/A
PCR Fragment V24.4: Amplified from plasmid pCBC-2 using primers V24.PromT7CimA.F & V24.CimA.R	This study	N/A
PCR Fragment P1.INSERT: Amplified from plasmid pCBC-3 using primers V24P1.CimA3.7.F & V24.CimA.R	This study	N/A
PCR Fragment V33.1: Amplified from plasmid pCBC-V24P1 using primers V33.Vec.1 & V33.Vec.2	This study	N/A
PCR Fragment V33.2: Amplified from plasmid E. coli BW25113 genomic DNA using primers V33.proB.F & V33.proA.R	This study	N/A
PCR Fragment V36.1: Amplified from plasmid pSTV28 using primers V36.CmR.F & V36.CmR.R	This study	N/A
PCR Fragment V36.2: Amplified from plasmid E. coli BW25113 gDNA using primers V36.ssb.F & V36.ssb.R	This study	N/A
PCR Fragment V36.3: Amplified from plasmid pCBC-V24P1 using primers V36.Vec.1 & V36V43.Vec.2	This study	N/A
PCR Fragment V43.1: Amplified from plasmid pCBC-V36 using primers V43.CmR.F & V43.CmR.R	This study	N/A
PCR Fragment V43.2: Amplified from plasmid pCBC-V24P1 using primers V43.Vec1 & V36V43.Vec2	This study	N/A
PCR Fragment proBA.KO: Amplified from gene-block gBlock-1 using primers proBA.KO.F & proBA.KO.R	This study	N/A
PCR Fragment ssb.KO: Amplified from gene-block gBlock -1using primers ssb.KO.F & ssb.KO.R	This study	N/A
PCR Fragment proC.KO: Amplified from gene-block gBlock -1using primers proC.KO.F & proC.KO.R	This study	N/A
PCR Fragment dapD.KO: Amplified from gene-block gBlock -1using primers dapD.KO.F & dapD.KO.R	This study	N/A
Plasmid: pKD46	Datsenko and Wanner^32^	https://doi.org/10.1073/pnas.120163297
Plasmid: pSTV28	Takara Bioscience	N/A
Plasmid: pCOLADuet™-1	MerckMillipore	N/A
Plasmid: pSC101	Miniprep from (DSM-6202)	N/A
Plasmid: pCBC-2	Yiakoumetti et al.^11^	https://doi.org/10.1016/j.isci.2025.111787
Plasmid: pCBC-3	Yiakoumetti et al.^11^	https://doi.org/10.1016/j.isci.2025.111787
Plasmid: pUC57:proC-3	This study	N/A
Plasmid: pUC57:dapD-3	This study	N/A
Plasmid: pCBC-V24, HiFi V24.1, V24.2, V24.3, V24.4	This study	N/A
Plasmid: pCBC-V24P1	This study	N/A
Plasmid: pCBC-V33, HiFi assembly of V33.1, V33.2	This study	N/A
Plasmid: pCBX-V36, HiFi assembly of V36.1, V36.2, V36.3	This study	N/A
Plasmid: pCBC-V43, HiFi assembly of V43.1, V43.2	This study	N/A
Plasmid: pCBC-V43:proC	This study	N/A
Plasmid: pCBC-V43:dapD	This study	N/A

**Software and algorithms**		

Multiple linear regression	MATLAB R2023a	Statistics and Machine Learning toolbox
Principal component analysis	MATLAB R2023a	Statistics and Machine Learning toolbox
Radial basis function neural network model: architecture & confidence limits	Leonard et al.^49^	https://doi.org/10.1016/0098-1354(92)80035-8
Radial basis function neural network reliability detection: architecture & membership density measure, Figure S1	This study	N/A
HPLC chromatogram analysis	Agilent ChemStation	N/A

### Experimental model and study participant details

#### Bacterial strains

All experimental work used *E. coli* BW25113 ΔldhA::FRT^11^ as the base strain. All modifications detalis are described in the plasmid and strain construction section.

### Method details

#### Materials and methods

##### Media

LB medium from Sigma was prepared according to the manufacturer’s protocol. For LB-agar plates, the LB was supplemented with 15 g L^−1^ agar. All starter culture and flask media were sterilised via autoclaving at 121°C for 20 min. For shake flasks, either Lund media or mineral salt (MS) media was used. For Lund media (per L): 2 g (NH_4_)_2_SO_4_, 14.6 g K_2_HPO_4_, 3.6 g NaH_2_PO_4_·H_2_O, 0.5 g (NH_4_)_2_H-citrate, 10 g glucose and 2 mL 500× trace element solution, then adjusted to pH 7 with HCl and filter sterilised. The 500× trace element solution contained (per L): 22.3 g Na_2_EDTA·2H_2_O, 0.5 g CaCl_2_·2H_2_O, 10.03 g FeCl_3_, 0.18 g ZnSO_4_·7H_2_O, 0.16 g CuSO_4_·5H_2_O, 0.15 g MnSO_4_·H_2_O, and 0.18 g CoCl_2_·6H_2_O. For MS media: MS Solution A (800 mL) containing 2 g KH_2_PO_4_, and 2 mL modified Vishniac trace elements, adjusted to pH 7; and MS Solution B (200 mL) containing 4 g NH_4_Cl and 0.4 g MgSO_4_·7H_2_O. The media was combined after autoclaving along with 20 mL of 250 g L^−1^ glucose stock solution and filter sterilised antibiotics as required in Table 4. The modified Vishniac trace elements (1L) contained: 50 g Na_2_EDTA dissolved in 800 mL distilled water and adjusted to pH 7 using KOH, followed by 2.2 g ZnSO_4_, 5.54 g CaCl_2_, 5.06 g MnCl_2_·4H_2_O, 5 g FeSO_4_.7H_2_O, 1.1 (NH_4_)_6_Mo_7_O_24_·4H_2_O, 1.57 g CuSO_4_·5H_2_O and 1.61 g CoCl_2_·6H_2_O. The final solution was adjusted to pH 6 and stored at 4°C.

For fermentation, phosphate-limiting MS medium was used. The media was prepared in 20 L volumes and filter sterilised using 0.2μm PES filters (Sartopore 2 300, Sartorius) into pre-autoclaved 20L bottles containing 5 mL polypropylene glycol 2000 (CAS 25322-69-4) as antifoam. The phosphate-limiting MS medium (20 L) contained: 1.36 g KH_2_PO_4_, 21.18 g KCl, 80 g NH_4_Cl, 8 g MgSO_4_·7H_2_O, 40 mL modified Vishniac trace elements and 320 g glucose. The tetracycline stabilised fermentations were supplemented with tetracycline, as per Table 4.

For patch-plating, MS flask media was supplemented with 15 g L^−1^ agar in MS Solution A. Additional filter sterilised supplements were added as specified in Table 4, depending on the strain being tested. Stock solutions of 10 g L^−1^ L-proline adjusted to pH 7 and 25 g L^−1^ LL-2,6-diaminopimelic acid (DAP) adjusted to pH 7 were used.

#### Plasmid and strain construction

All strains and plasmids utilized and constructed in this study are described in the key resources table, with further plasmid details in Table S1. Details of all oligonucleotides, PCR and HiFi reactions, enzymes, molecular biology master mixes and kits referenced in this study are provided in the key resources table and Table S2-S3. Details of construction for strain CBC-infA, and plasmids pCBC-2 and pCBC-3 provided in Yiakoumetti et al*.*^11^

Plasmids pCBC-V24, pCBC-V33, pCBC-V36 and pCBC-V43 were assembled by HiFi assembly (key resources table) using the NEBuilder HiFi DNA Assembly Master Mix as per the manufacturer’s protocol and using a 60 min incubation time. Fragments for the assemblies were all generated by PCR reactions (Table S1). To construct plasmid pCBC-V24P1: PCR fragment P1.INSERT (Table S1), encoding cimA3.7 and the J23119 promoter, was digested with *Bam*HI and *Sbf*I, and the 1.2 kbp fragment was ligated into a 2.45 kbp fragment resulting from the digestion of pCBC-V24 with *Bgl*II and *Sbf*I. Assembly of pCBC-V43:proC and pCBC-V43:dapD was performed by GoldenGate assembly, using pCBC-V43 as the recipient vector, pUC57:proC-3 as the donor vector for the assembly of pCBC-V43:proC, and pUC57:dapD as the donor vector for the assembly of pCBC-V43:dapD. GoldenGate reactions (20 μL) were composed of recipient vector (20 fmol), donor vector (40 fmol), *Sap*I (1 unit), T4 DNA ligase (1 unit), and buffer for T4 DNA ligase (1X). Assembly reactions were performed in a thermocycler, starting with 30 cycles of 5 min at 37°C and 5 min at 16°C, followed by a further 5 min incubation step at 37°C and a heat inactivation step at 65°C for 20 min. All plasmids assembled as per the above methodologies were transformed into NEB 5-alpha chemically competent cells by heat shock, as per the manufacturer’s protocol. High Efficiency competent cells were used for pCBC-V24 assembled by 4-part HiFi assembly, and sub-cloning efficiency cells for all other plasmids. Transformed cells were plated onto LB-agar supplemented with the appropriate antibiotics, i.e., 12 μg mL^−1^ tetracycline for cells transformed with pCBC-V24, pCBC-V24P1 and pCBC-V33, and 34 μg mL^−1^ chloramphenicol for cells transformed with pCBC-V36, pCBC-V43, pCBC-V43:proC and pCBC-V43:dapD. Mini-preps (QIAprep Spin Miniprep kit, Qiagen) of plasmids were undertaken from overnight cultures, cultivated in 5 mL LB with appropriate antibiotic at 37°C, 250 rpm for approximately 16 h. Plasmid sequences were verified by Sanger Sequencing.

To construct the tetracycline stabilised control strain CBC-tet, plasmid pCBC-V24 was transformed into *E. coli* BW25113 Δ*ldhA*::FRT, and strain maintenance was performed in LB media supplemented with 12 mg mL^−1^ tetracycline. Gene knockouts for strains stabilised by plasmid addiction systems were performed by Lambda-RED recombineering.^55^ PCR amplification of a spectinomycin resistance marker was used to generate PCR products proBA.KO, ssb.KO, proC.KO and dapD.KO (Table S1) with ∼50 bp homologous arms for the deletion of *proBA*, *ssb*, *proC* and *dapD*, respectively. For the deletion of *proBA* and *proC*, electro-competent *E. coli* BW25113 Δ*ldhA*::FRT cells were prepared and transformed with pKD46. Upon transformation, outgrowth was performed at 30°C and 250 rpm for 3 h before plating on LB agar supplemented with carbenicillin (100 mg L^−1^), and incubated at 30°C for 24 h. An overnight culture (10 mL LB supplemented with 100 mg L^−1^ carbenicillin) was inoculated from a single colony, and incubated at 30°C, 250 rpm for 16 h. Thereafter, 1 mL of overnight culture was inoculated into 100 mL LB supplemented with carbenicillin (100 mg L^−1^) and L-arabinose (10 mL) for 6–8 h (day culture). The day culture was incubated at 30°C and 250 rpm until OD_600_ 0.3–0.4, then chilled on ice for 10 min. Cells were harvested by centrifugation (2000 × *g*, 4°C, 10 min) and re-suspended in 100 mL ice-cold sterile water. Cell harvesting was repeated thrice more, re-suspending once in 10 mL ice-cold 10% (v/v) glycerol, then in 1 mL ice-cold 10% (v/v) glycerol, and finally in 100 μL ice-cold 10% (v/v) glycerol. Electro-competent cells (40 mL) were mixed with 500 ng PCR product, encoding the knockout cassette in pre-chilled 2 mm gap electroporation cuvette, and electroporation was performed at 2.5 kV, 200 Ω and 25 μF. Outgrowth was performed in 1 mL SOC medium supplemented with 10 mM L-arabinose, and at 30°C and 250 rpm for 3 h. Cells were harvested by centrifugation (5000 rpm, 10 min) and re-suspended in 100 μL SOC medium (lacking L-arabinose), before all the cells were plated on LB agar plates supplemented with 50 μg mL^−1^ spectinomycin and pre-warmed to 37°C. Plates were incubated overnight at 37°C. For the deletion of *dapD*, all cell culturing steps from the 100 mL day culture were performed with additional supplementation of 250 mg L^−1^ DAP. I.e. the day culture was performed in 100 mL LB supplemented with 100 μg mL^−1^ carbenicillin, 10 mM L-arabinose and 250 mg L^−1^ DAP; after electroporation, transformants were propagated in SOC medium supplemented with 10 mM L-arabinose and 250 mg L^−1^ DAP for outgrowth; after outgrowth, cells were harvested and resuspended in 100 μL of SOC medium supplemented with 250 μg mL^−1^ DAP; and cells were plated on LB agar plates supplemented with 50 μg mL^−1^ spectinomycin and 250 μg mL^−1^ DAP. *E. coli* BW25113 *ΔldhA*::FRT Δ*dapD*::*sp*^R^ was maintained in LB medium supplemented with 250 μg mL^−1^ DAP. Finally, the deletion of *ssb* was performed as for *proBA* and *proC*, but with plasmid pCBC-V36 *in situ*, such that plasmid pCBC-V36 was co-transformed along with plasmid pKD46 into *E. coli* BW25113 Δ*ldhA*::FRT. Therefore, this necessitated the inclusion of both 100 μg mL^−1^ carbenicillin and 34 μg mL^−1^ chloramphenicol for the maintenance of *E. coli* BW25113 Δ*ldhA*::FRT pKD46 pCBC-V36 on LB agar plates, in the LB seed culture, and in the LB day culture also supplemented with L-arabinose. Chloramphenicol supplementation was not required after successful deletion of *ssb*.

After the curing of plasmid pKD46 at 37°C, the deletion of the *ssb* in the presence of pCBC-V36 resulted in the final strain CBC-ssb, where maintenance of this strain did not require any antibiotic supplementation in either LB medium or minimal medium. To construct strains CBC-proBA and CBC-proC, *E. coli* BW25113 Δ*ldhA*::FRT Δ*proBA*::*sp*^R^ and *E. coli* BW25113 Δ*ldhA*::FRT Δ*proC*::*sp*^R^ were respectively transformed with pCBC-V33 and pCBC-V43:proC by standard protocols for competent cell preparation and transformation. CBC-proBA was plated on LB agar supplemented with 12 μg mL^−1^ tetracycline, and CBC-proC was plated on LB agar supplemented with 34 μg mL^−1^ chloramphenicol. Subsequent maintenance of strains CBC-proBA and CBC-proC required antibiotic supplementation in LB media, but did not require antibiotic supplementation in minimal media. To construct CBC-dapD, competent cells of *E. coli* BW25113 Δ*ldhA*::FRT Δ*dapD*::*sp*^R^ were prepared with 250 μg mL^−1^ DAP supplementation at all stages of cell growth. Upon transformation with pCBC-V43:*dapD*, outgrowth was performed in SOC medium supplemented with 250 μg mL^−1^ DAP before plating on LB agar supplemented with 34 μg mL^−1^ chloramphenicol. Subsequent maintenance of CBC-dapD in LB media did not require the supplementation of either DAP or chloramphenicol.

#### Strain validation

All strains were tested in shake flasks using Lund media for growth and assessment of citramalate production, prior to evaluation in fermentation (data not presented).

#### Continuous fermentation

Fermentations were carried out in 1.3 L (0.7 L or 0.9 L working volume for dilution rates of 0.1 h^−1^ and 0.033 h^−1^, respectively) or 3 L (2 L working volume) BioFlo 115 bioreactors (Eppendorf). The fermentations were cultivated at 30°C or 37°C, with an aeration rate of 1 vvm. The pH was controlled at 7.0 via the addition of 3 M KOH. The agitation speed was fixed at 450 rpm for the 1.3 L bioreactors and at 500 rpm for the 3 L bioreactors. This ensured the dissolved oxygen concentration was maintained above 30% throughout the fermentation. The volume was fixed via a harvest tube connected to a peristaltic pump. Fresh feed was fed into the fermenter via a peristaltic pump maintaining a dilution rate (D = F/V) of either 0.033 h^−1^ or 0.1 h^−1^ based on the working volume of ungassed fermentation broth. Watson Marlow 120U pumps were used for both the feed and harvest with 0.8 mm and 3.2 mm internal diameter Marprene tubing, respectively. Samples were taken through an aseptic sampling port on the fermenter.

For fermentation, all inocula stemmed from a single colony, cultivated overnight (∼16 h) at 30°C on LB agar plates supplemented with the appropriate antibiotic as per Table 4. A single colony was transferred to 10 mL LB, supplemented with the appropriate antibiotic, and cultivated for 12 to 16 h at 30°C and 250 rpm. This starter culture was transferred to 50 mL or 100 mL MS media, diluted to an initial OD_600_ of 0.1. The MS flask was cultivated for 5 to 7 h at the selected fermentation temperature and shaker speed of 250 rpm to attain a mid-exponential phase OD_600_ of 1.3. The MS flask was used to inoculate the fermenter, the volume used varied to achieve a starting OD_600_ of 0.075. The continuous feed was started once the culture had reached exponential phase, i.e., once attaining an OD_600_ of approximately 1. For the dilution rate of 0.033 h^−1^, an initial dilution rate of 0.05 h^−1^ was used for the first 12 to 16 h, thereby ensuring that the fermentation avoided glucose limitation owing to any excess phosphate transferred to the fermenter from the inoculum.

#### Fermentation parameters

The following parameters were calculated to assess the fermentation performance:

$$Specific\;productivity=C_{CMA}\cdot D$$

$$Specific\;uptake\;rate=C_{Glu\;consumed}\cdot D$$

Yield(PS)=CCMACGluconsumed

Yield(PX)=CCMACBiomass

Yield(XS)=CBiomassCGluconsumed

### Quantification and statistical analysis

#### Analytical methods

The cell density (OD_600_) was measured via UV-vis spectrophotometry at 600 nm. The samples were diluted with deionized water if the OD was greater than 0.6. This was correlated with the dry weight of 0.34 g L^−1^ per OD_600_.^11^

Citramalic acid, acetic acid and glucose concentration were measured using an Agilent 1200 Infinity series HPLC equipped with an UV-detector set to 210 nm and refractive index detector (RID). A Rezex ROA-Organic acid H+ (8%) 30 × 4.6 mm column (Phenomenex) was run at 55°C using a 0.01 N H_2_SO_4_ mobile phase at 0.5 mL min^−1^, maintaining the RID at a constant temperature of 35°C. The chromatogram was integrated using ChemStation software. A calibration curve was established using standards ranging between 0.375 and 20 g L^−1^ for glucose, 0.375 and 5 g L^−1^ for acetic acid, and 0.313 and 10 g L^−1^ for citramalic acid.

The fermentation samples were prepared by centrifuging at 8000 rpm for 10 min, followed by filtration of the supernatant using a 0.2μm regenerated cellulose filter.

#### Segregational stability

A patch-plating method was employed to assess segregational stability, using non-selective and selective plate media as specified in Table 4. This method has been widely used to test plasmid segregational stability.^12^^,^^13^^,^^14^^,^^17^ The non-selective plates were the same base media as the fermentation media, either with no antibiotic supplemented or the essential gene product supplemented depending on the addiction system tested. The monoseptic fermentation sample was serially diluted using sterile 5 mM MgCl_2_, spread on non-selective plates and incubated at the fermentation temperature at 37°C and 30°C for 24 or 48 h respectively. Fifty colonies were picked and transferred to both selective and non-selective plates and incubated at the fermentation temperature for 24–48 h. The ratio of colonies that grew on both plates served as indication of segregational stability.

#### Structural stability

Structural instability was indirectly inferred when a decrease in productivity was observed and the plasmid was maintained in the cell, as demonstrated by the patch plating.

#### Multivariate data analysis

Multivariate data analysis was performed using the average steady state fermentation data. Three exploratory data analysis methods were performed: principal component analysis (PCA), multiple linear regression (MLR) and non-linear regression through radial basis function neural networks (RBF).

The PCA and MLR were undertaken using the Statistics and Machine Learning toolbox from MATLAB R2023a. For the MLR, the data were randomly partitioned into a 70% training set and 30% test set. Confidence limits for the MLR were calculated using the residual standard error and the two tailed inverse of the Student’s t-distribution for the degrees of freedom at significance level 0.05.

For the RBF, the architecture devised by Leonard et al.^49^ was implemented, incorporating both confidence limits and reliability detection as a measure of statistical significance for the non-linear model prediction. Figure S1 (Supplementary information) summarizes the architecture for the non-linear regression using radial basis function neural networks. The confidence limit for each RBF, *CL*_*h*_, is a function of the sum square error and the two tailed inverse of the Student’s t-distribution for the degrees of freedom at significance level 0.05.

This work modifies the original concept in Leonard et al. (1992), redefining the membership density measure as in Equation 1, where $\rho_{h}$ is the density measure for each radial basis function, k is the index of an input vector, K is the total number of input vectors and $a_{h}$ is the activation for input vector $x_{k}$.

(Equation 1) $$\rho_{h}=\frac{\sum_{k=1}^{K}a_{h,k}}{K}$$

Thereby, $\rho_{h}$ has a minimum value of zero and a maximum value of one. Similarly, the density measure for each individual input vector across the RBF network is calculated using Equation 2, where $\rho \left(x_{k}\right)$ is the density measure for each input vector, h is the hidden node index and H is the number of hidden nodes. Similar to $\rho_{h}$, $\rho \left(x_{k}\right)$ has a value ranging between zero and one, depending on the input vector’s respective membership of each RBF node.

(Equation 2) $$\rho \left(x_{k}\right)=\frac{\sum_{h=1}^{H}a_{h,k}\cdot \rho_{h}}{\sum_{h=1}^{H}\rho_{h}}$$

A reliability RBFNN model was constructed to predict the density measure for the training set using the density measure calculated for each input vector as defined by Equation 2. This reliability model sits adjacent to the predictive model (Figure S1), accepting the same input vectors, thereby predicting the density measure and its confidence limits. This reliability measure is used to validate the predictive model’s output, thereby ensuring that predictions are underpinned by training data or otherwise flagged as extrapolation. This is important because the RBF will always provide a predicted output within the confidence limits provided by the training set, and therefore, with confidence limits alone it is not possible to definitively deduce whether the RBFNN is extrapolating. Extrapolation is defined as a predictive output using an input vector sparsely represented within the training set of the RBFNN, meaning the unseen input vector does not have predictable membership of the network’s radial basis functions. To overcome this uncertainty, the modified reliability measure assesses the predictability of an input vector’s membership. Should the reliability model prediction fall within its confidence limits, provided by the training set, this indicates that the predictive model’s output (confidence limits) has greater certainty, i.e., within the lower and upper bounds, the predictive model’s output is produced by the phenomenon or mechanism as modeled by the RBF network.
