# Supplementary material for: Enhancing the stability of continuous fermentations for platform chemical production
Source: iScience. 2025 Jan 30;28(3):111786. doi: 10.1016/j.isci.2025.111786 (PMC11875142; doi:10.1016/j.isci.2025.111786)
Supplement: Document S1. Figures S1, S2, and Tables S1–S4 [file mmc1.pdf]

iScience, Volume *28*

Supplemental information

Enhancing the stability of continuous fermentations for platform chemical production

Victoria Outram, Andrew Yiakoumetti, Charlotte Green, Rebekah King, John M. Ward, and Alex Conradie

Supplementary information

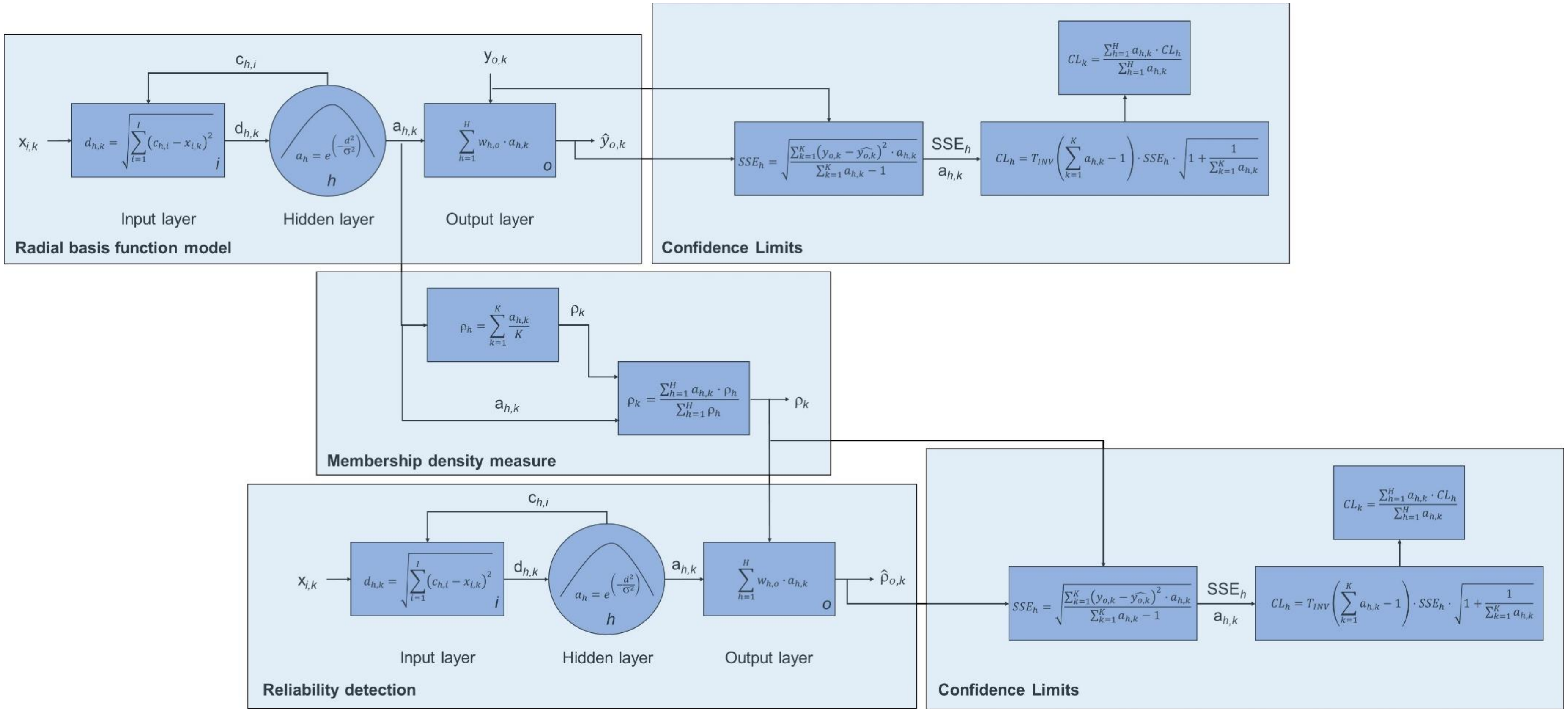

Figure S1: Radial Basis Function Network (RBF) architecture incorporating confidence limits and reliability detection. The localised mapping of input to output space by radial basis functions provides for implicit membership assignment of input vectors to each RBF node in the network, facilitating both confidence limit and reliability detection, where $x_{i,k}$ is the input vector's value at dimension, i, for observation k; $c_{h,i}$ is the centre point value of the RBF node, h, at dimension, i; $d_{h,k}$ is the Euclidean distance between the centre point, $c_{h,i}$, and input vector $x_{i,k}$; a_h is the activation value of RBF node, h; σ is the spread of RBF node, h; $w_{h,o}$ is the weighting for RBF h's activation, a_h, to the output vector at dimension, o. The summation of the hidden layer's weighted RBF outputs produces the predictive output, $\widehat{y_{o,k}}$, for the observation, $y_{o,k}$. Each RBF, h, bears a weighted burden of the sum squared error, SSE_h, as a proportion of its activation across the observations. The confidence limit for each RBF, CL_h, is a function of the two tailed inverse of the Student's t-distribution for the degrees of freedom at significance level 0.05 and the SSE_h. Finally, the confidence limit for observation k, CL_k, is the weighted sum of each RBF's CL_h. The membership density for each RBF, h, is defined as ρ_h, where the membership density for the observation, k, is the weighted summation of each ρ_h, defined as ρ_k. Related to STAR methods.

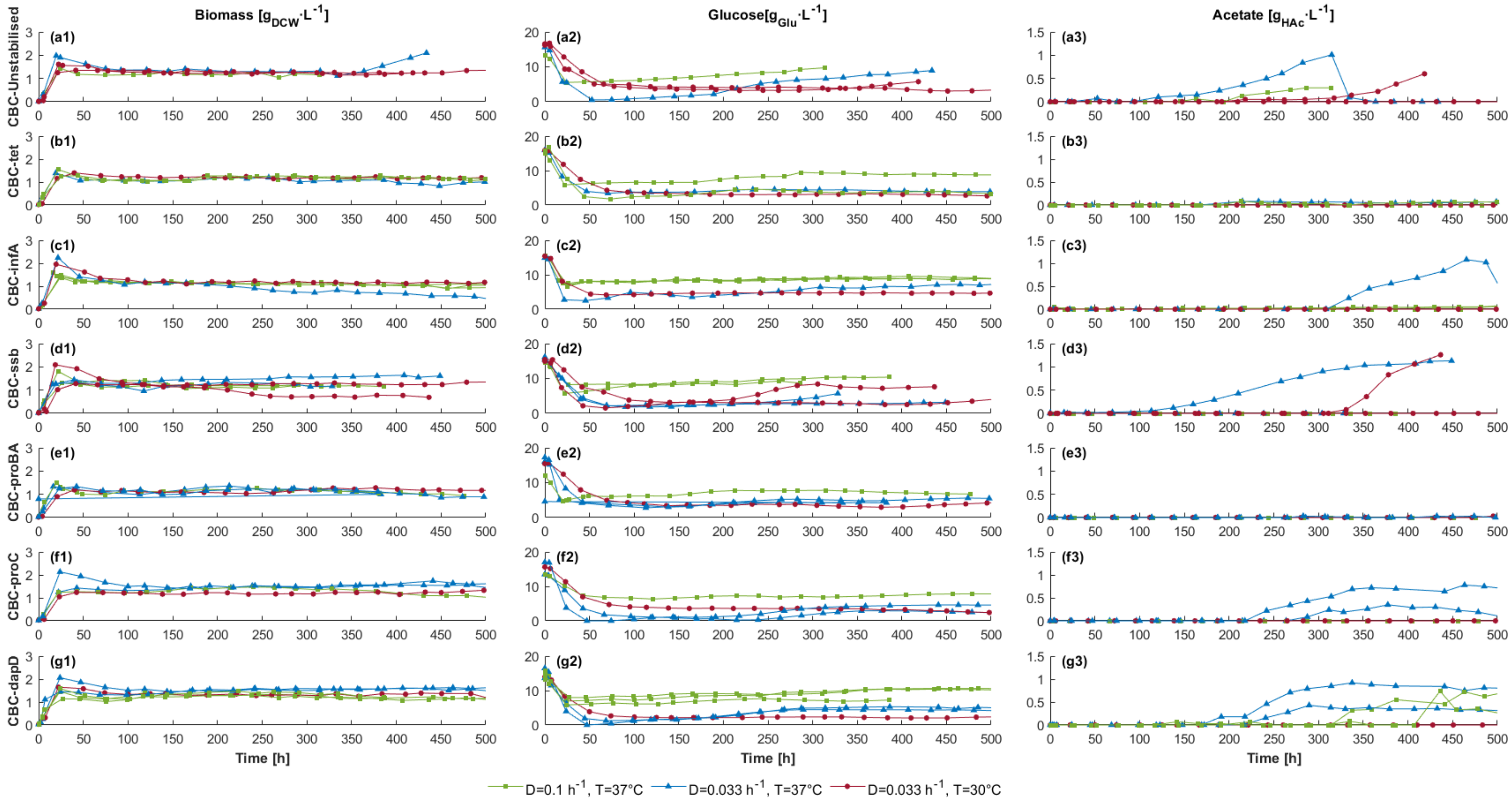

Figure S 2: Biomass, glucose and acetate concentration data during continuous fermentations evaluated for the five test strains and two benchmark strains at three operating conditions. Red diamonds represent operation at D=0.033 h^{-1} and T=30 °C. Blue upward triangles represent operation at D=0.033 h^{-1} and T=37 °C. Green squares represent operation at D=0.1 h^{-1} and T=37 °C. Each line represents one fermentation at that condition, repeats were performed to confirm the observed instability. The columns detail the citramalate concentration, specific productivity, product yield and % plasmid-bearing cells, respectively. The rows represent the fermentations for each of the strains in the order of unstabilised strain, tetracycline stabilised, infA stabilised, ssb stabilised, proBA stabilised, proC stabilised and dapD stabilised, respectively. This data compliments the data presented in Figure 1.

Table S1: Plasmids constructed in this work and their purpose. Related to STAR methods.

Plasmid	Purpose	Source
pKD46	Lambda RED helper plasmid	[49]
pSTV28	Template for chloramphenicol resistance marker	Takara Bioscience
pCOLADuet™-1	Template for pCOlA origin of replication	MerckMillipore
pSC101	Template for tetracycline resistance marker	Miniprep from (DSM-6202)
pCBC-2	Template for *cimA*3.7 with T7 promoter	[32]
pCBC-3	Template for *cimA*3.7 with J23119 promoter	[32]
pUC57::proC-3	Donor vector for assembly of pCBC-V23::*proC*	Custom Synthesis, Genscript
pUC57::dapD-3	Donor vector for assembly of pCBC-V23::*dapD*	Custom Synthesis, Genscript
pCBC-V24	Tetracycline stabilised intermediate plasmid for the construction of pCBC-V24P1	This study, Sequence S1
pCBC-V24P1	Tetracycline stabilised plasmid for expression of *cimA3.7*	This study, Sequence S1
pCBC-V33	proBA stabilised plasmid for the expression of *cimA3.7*	This study
pCBC-V36	ssb stabilised plasmid for the expression of *cimA3.7*	This study
pCBC-V43	Recipient vector for proC and dapD stabilisation cassettes – Expresses *cimA3.7*	This study
pCBC-V43::proC	proC stabilised plasmid for the expression of *cimA3.7*	This study
pCBC-V43::dapD	dapD stabilised plasmid for the expression of *cimA3.7*	This study

Table S2: PCR reactions performed in this study. All PCR reactions were performed using Q5 DNA polymerase, as per the manufacturer's protocol. Related to STAR methods.

PCR Fragment ID	Template	Primer 1	Primer 2
V24.1	pCOLADuet-1	**V24.oriColA.F**	**V24.oriColA.R**
V24.2	pCOLADuet-1	V24.ColATermT7.F	V24.ColATermT7.R
V24.3	pSC101	V24.TcR.F	V24.TcR.R
V24.4	pCBC-2	V24.PromT7CimA.F	V24.CimA.R
P1.INSERT	pCBC-3	V24P1.CimA3.7.F	V24.CimA.R
V33.1	pCBC-V24P1	V33.Vec.1	V33.Vec.2
V33.2	*E. coli* BW25113 gDNA	V33.proB.F	V33.proA.R
V36.1	pSTV28	V36.CmR.F	V36.CmR.R
V36.2	*E. coli* BW25113 gDNA	V36.ssb.F	V36.ssb.R
V36.3	pCBC-V24P1	V36.Vec.1	V36V43.Vec.2
V43.1	pCBC-V36	V43.CmR.F	V43.CmR.R
V43.2	pCBC-V24P1	V43.Vec1	V36V43.Vec2
proBA.KO	Gblock-1, Table S4	proBA.KO.F	proBA.KO.R
ssb.KO	pIJ778	ssb.KO.F	ssb.KO.R
proC.KO	pIJ778	proC.KO.F	proC.KO.R
dapD.KO	pIJ778	dapD.KO.F	dapD.KO.R

Table S3: HiFi reactions performed in this study. HiFi reactions were performed using NEBuilder® HiFi DNA Assembly cloning kit. Related to STAR methods.

Plasmid assembled by HiFi assembly	Parts in assembly	Part ID
pCBC-V24	4	V24.1
		V24.2
		V24.3
		V24.4
pCBC-V33	2	V33.1
		V33.2
pCBC-V36	3	V36.1
		V36.2
		V36.3
pCBC-V43	2	V43.1
		V43.2

Table S4: Oligonucleotides used in this study. Related to STAR methods.

Oligonucleotides	Identifier in this study
Primer: CCAAACGATAAAGGGTGATGTGTTTACTGATATGAAAAGAGTTTAACAATACTATTTGCAACAGTGCC	dapD.KO.F
Primer: CTCATCCCTGCAAAAAAATCGGGCAGCGTCGTGCTGCCCGTGTGCATACTTTTATTTGCCGACTACCTTGGTG	dapD.KO.R
Primer: GGCAACCGACGACAGTCCTGCTAAAACGTTCGTTTGATATCATTTTTCCTACTATTTGCAACAGTGCC	proBA.KO.F
Primer: GTCAATGGCCTTGTGAATCAAATGGCTACTTTTGCATCACCCGGTTTTATTTATTTGCCGACTACCTTGG	proBA.KO.R
Primer: GCTTCTGCCAGCGATTATCAAAACAATGAATTTCACGGCAGGAGTGAGGCAACTATTTGCAACAGTGCCG	proC.KO.F
Primer: GCCGGACGTAACCGCACCGAAGTGGCGGCCTGACGTCCGGCGAAAGTCATTATTTGCCGACTACCTTGGTG	proC.KO.R
Primer: ACCTGAATGAATATACAGTATTGGAATGCATTACCCGGAGTGTTGTGTAAACTATTTGCAACAGTGCC	ssb.KO.F
Primer: GCTTTAATCATCCACCTTAAAACAATATAACCTATTGTTTTAATGACAAATTATTTGCCGACTACCTTGG	ssb.KO.R
Primer: AGCAGCCTAGGTTAACCTGCAGGTTACAGTTTACCAGTAACCTC	V24.CimA.R
Primer: ACTGTAACCTGCAGGTTAACCTAGGCTGCTGC	V24.ColATermT7.F
Primer: ACCTCGACCTGAGAATTAATTCATGAGCGGATACATATTTG	V24.ColATermT7.R

Primer: GAGTCGTATTAAGATCTGCGCAACGCAATTAATGTAAGTTAGCTC	V24.oriColA.F
V24.oriColA.R: TTATCGATGATAAGCTGTCAAACATGAGAATTTAGGCATGCTAGCGCAGAAACG	V24.oriColA.R
Primer: CATTAATTGCGTTGCGCAGATCTTAATACGACTCACTATAGCTCGAG	V24.PromT7CimA.F
Primer: AATTCTCATGTTTGACAGCTTATCATCGATAAGC	V24.TcR.F
Primer: CCGCTCATGAATTAATTCTCAGGTCGAGGTGGCCC	V24.TcR.R
V24P1.CimA3.7.F: CGTCAAGCCGTCAATTGTCTGATTCGTTACC	V24P1.CimA3.7.F
Primer: GCTCATGAATTAATTCTTACGCACGAATGGTGTAATCACC	V33.proA.R
Primer: GCTTATCATCGATAAGCTTTAATGCGGTAGTTTATCACAGTTAAATTGCAAAATTGAATGGCAGAGAATCATGAGTGAC	V33.proB.F
Primer: CCATTCGTGCGTAAGAATTAATTCATGAGCGGATACATATTTGAATG	V33.Vec.1
Primer: GCATTAAAGCTTATCGATGATAAGCTGTCAAACATGAGAATTTATCAGGTCGAGGTGGCCC	V33.Vec.2
Primer: CTGATTTGTGCGGCCGCAATTCTCATGTTGATCGGCACGTAAGAGG	V36.CmR.F
Primer: GTATCCGCTCATGAATTAATTCGCGGCCGCTTACGCCCCGCCCTGCC	V36.CmR.R
Primer: GCTAGCATGCCTACAATGTCTGGCCAGGTTTG	V36.ssb.F
Primer: CATGAGAATTGCGGCCGCACAAATCAGAACGGAATGTCATCATC	V36.ssb.R
Primer: CCTGGCCAGACATTGTAGGCATGCTAGCGCAG	V36.Vec.1
Primer: GCGGCCGCGAATTAATTCATGAGCGGATACATATTTGAATG	V36V43.Vec.2
Primer: CAGTAGGTTATCACAGTCAACTTAAGCTCTTCATAAGCGGCCGCAATTCTCATGTTGATC	V43.CmR.F
Primer: GTATCCGCTCATGAATTAATTCGCGGCCGCTTACGCCCCGCCCTGCC	V43.CmR.R
Primer: TTAAGTTGACTGTGATAACCTACTGGCTCTTCTCTGTAGGCATGCTAGCGCAGAAACGTC	V43.Vec1
